# The Northeast Chinese species of *Psathyrella* (Agaricales, Psathyrellaceae)

**DOI:** 10.3897/mycokeys.33.24704

**Published:** 2018-04-13

**Authors:** Jun-Qing Yan, Tolgor Bau

**Affiliations:** 1 Engineering Research Centre of Chinese Ministry of Education for Edible and Medicinal Fungi,; 2 Jilin Agricultural University,; 3 Changchun 130118, P. R. China

**Keywords:** Basidiomycete, new taxon, phylogenetic analysis, taxonomy

## Abstract

Twenty seven species of *Psathyrella* have been found in Northeast China. Amongst them, *P. conica*, *P. jilinensis*, *P. mycenoides* and *P. subsingeri* are described as new species, based on studying morphological characteristics and phylogenetic analyses. Detailed morphological descriptions, line drawings and photographs of the new species are presented. Phylogenetic analysis of the nuclear ribosomal internal transcribed spacer (ITS) region and an identification key to the 27 *Psathyrella* species occuring in Northeast China are provided.

## Introduction

*Psathyrella* (Fr.) Quél. is one of the large genera of Agaricales Underw. which consists of 1,030 records in Index Fungorum (http://www.indexfungorum.org), comprising approximately 500 species (Smith 1972; Kits van Waveren 1985; Örstadius and Kundsen 2012;). It is characteristic of fragile basidiomata, hygrophanous pileus, brown to black brown spore print, always present cheilocystidia and basidiospores smooth or rarely granulose or with myxosporium, fading to greyish in concentrated sulphuric acid (H_2_SO_4_).

The studies of this genus mainly focused on Europe and North America in recent years (Romagnesi 1952; Smith 1972; Kits van Waveren 1985; Nagy et al. 2011; Örstadius and Kundsen 2012; Örstadius et al. 2015). In China, 51 names (*Psathyrella* s.l.) were reported, including four new species (Chiu 1973; Bi et al. 1985; Bi et al. 1987; Bi 1991; Wang and Bau 2014). Amongst them, 21 species can be found in Northeast China which includes Helongjiang Province, Jinlin Province, Liaoning Province and the northeast of Inner Mongolia Autonomous Region (Wang 2014).

Due to the morphological plasticity of the *Psathyrella*, some species cannot be distinguished clearly and many names have been combined (Örstadius and Kundsen 2012). Therefore, the aim of this study is to clarify the diversity of *Psathyrella* in Northeast China by traditional taxonomy and molecular phylogenetic analysis. The examined specimens (from 1997 to 2017) are deposited in the Herbarium of Mycology, Jilin Agricultural University (HMJAU). As a result of morphological and molecular observations, 27 species of *Psathyrella* were identified, and of which *P. conica*, *P. jilinensis*, *P. mycenoides* and *P. subsingeri* were reported as new species. Molecular phylogenetic affinities of the 27 species based on the nuclear ribosomal internal transcribed spacer (ITS) region and an identification key to them are provided.

## Materials and methods

### Morphological studies

Specimens are deposited in the Herbarium of Mycology, Jilin Agricultural University (HMJAU). Macroscopic characteristics were recorded from fresh specimens. Colour codes are from Kornerup and Wanscher (1978). Samples for microscopic examination were mounted in water and 5% aqueous KOH. Amyloid reactions were diagnosed in Melzer’s reagent. Thirty basidiospores, cystidia and basidia were measured for each collection. The basidiospores quotient (Q=L/B) was calculated from measurements of basidiospores.

### DNA extraction and sequencing

The NuClean Plant Genomic DNA kit (CWBIO) was employed for DNA extraction and PCR amplification from dried specimens. PCR was performed using a touchdown programme (Yan and Bau 2017) and the ITS region was amplified with the primer pair ITS1 and ITS4 (White et al. 1990). The details of sequenced specimens are given in Table 1. The DNA sequencing was done by Comate Bioscience Co., Ltd., Changcun City, China.

**Table 1.** Sequenced specimens used in phylogenetic analysis.

Taxa	Voucher	Locality	GenBank accession no. (ITS)
*P. amaura* (Berk. & Broome) Pegler	HMJAU 37810	Jilin: Qiupi Village, Tonghua City	MG734724
*P. bipellis* (Quél.) A.H. Sm.	HMJAU 25349	Jilin: Jilin Agricultural University	MG734722
*P. borealis*	HMJAU 37924	Inner Mongolia Autonomous Region: Mangui Town	MG734743
*P. borealis* A.H. Sm.	HMJAU 37911	Jilin: Changbai Mountain National Nature Reserve	MG734746
*P. boreifasciculata* Kytöv. & Liimat.	HMJAU 27556	Heilongjiang: Nanwenghe National Nature Reserve	KX901850
*P. candolleana*	HMJAU 37994	Jilin: Dayangcha, Erdaobaihe Town	MG734719
*P. candolleana* (Fr.) Maire	HMJAU 37994	Liaoning: Wulong Mountain	MG734720
*P. conica*	HMJAU 22096	Jilin: Lushuihe Town, Baishan City	MG734713
*P. conica*	HMJAU 37846 Type	Jilin: Changbai Mountain National Nature Reserve	MG734739
*P. conica*	HMJAU 37905	Jilin: Changbai Mountain National Nature Reserve	MG734745
*P. effibulata* Örstadius & E. Ludw.	HMJAU 37832	Jilin: Jingyuetan National Scenic Area	MG734727
*P. fennoscandica* Örstadius & E. Larss.	HMJAU 37918	Heilongjiang: Shuanghe National Nature Reserve	MG734723
*P. gordonii*	HMJAU 35984	Jilin: Jilin Agricultural University	KX901852
*P. gordonii* (Berk. & Broome) A. Pearson & Dennis	HMJAU 35983	Jilin: Jilin Agricultural University	KY120974
*P. jilinensis*	HMJAU 37822 Type	Jilin: Changbai Mountain National Nature Reserve	MG734717
*P. jilinensis*	HMJAU 37824	Jilin: Changbai Mountain National Nature Reserve	MG734721
*P. lutensis* (Romagn.) M.M. Moser	HMJAU 37840	Inner Mongolia Autonomous Region: Huihe National Nature Reserve	MG734748
*P. luteopallida* A.H. Sm.	HMJAU 5148	Jilin: Zuojia Town, Jilin City	MG734736
*P. mammifera*	HMJAU 21908	Jilin: Mahutou Mountain, Changchun City	MG734734
*P. mammifera* (Romagn.) Courtec.	HMJAU 37882	Jilin: Changbai Mountain National Nature Reserve	MG734740
*P. mycenoides*	HMJAU 37888 Type	Jilin: Jilin Agricultural University	MG734730
*P. mycenoides*	HMJAU 37993	Jilin: Jilin Agricultural University	MG734731
*P. obtusata*	HMJAU 37307	Jilin: Changbai Mountain National Nature Reserve	KY224080
*P. obtusata* (Pers.) A.H. Sm.	HMJAU 37310	Jilin: Changbai Mountain National Nature Reserve	KY224081
*P. panaeoloides* (Maire) Arnolds	HMJAU 23696	Jilin: Lushuihe Town, Baishan City	MG734733
*P. pertinax* (Fr.) Örstadius	HMJAU 6830	Jilin: Changbai Mountain National Nature Reserve	MG734735
*P. phegophila*	HMJAU 37848	Jilin: Songjiang Town	MG734738
*P. phegophila*	HMJAU 37804	Heilongjiang: Shengshan National Nature Reserve	MG734726
*P. phegophila* Romagn.	HMJAU 28267	Inner Mongolia Autonomous Region: Baiyin’aobao National Nature Reserve	MG734728
*P. piluliformis* (Bull.) P.D. Orton	HMJAU 37922	Heilongjiang: Shuanghe National Nature Reserve	MG734716
*P. pygmaea* (Bull.) Singer	HMJAU 37850	Jilin: Changbai Mountain National Nature Reserve	MG734744
*P. senex* (Peck) A.H. Sm.	HMJAU 4450	Inner Mongolia Autonomous Region: Hulunbeier City	MG734732
*P. singeri* A.H. Sm.	HMJUA 37867	Jilin: Changbai Mountain National Nature Reserve	MG734718
*P. spintrigeroides*	HMJAU 37820	Jilin: Changbai Mountain National Nature Reserve	MG367203
*P. spintrigeroides* P.D. Orton	HMJAU 37901	Jilin: Changbai Mountain National Nature Reserve	MG734737
*P. squamosa*	HMJAU 37816	Heilongjiang: Nanwenghe National Nature Reserve	MG367206
*P. squamosa* (P. Karst.) A.H. Sm.	HMJAU 35923	Jilin: Lushuihe Town, Baishan City	MG734729
*P. subsingeri*	HMJAU 37814	Yunnan: Yeya Lake	MG734714
*P. subsingeri*	HMJAU 37811	Jilin: Jilin Agricultural University	MG734715
*P. subsingeri*	HMJAU 37913 Type	Jilin: ingyuetan National Scenic Area	MG734725
*P. subsingeri*	HMJAU 37915	Henan: Boerdeng National Forest Park	MG734742
*P. subspadiceogrisea*	HMJAU 35992 Type	Jilin: Changbai Mountain National Nature Reserve	KY678465
*P. subspadiceogrisea* T. Bau & J.Q. Yan	HMJAU 35996	Jilin: Changbai Mountain National Nature Reserve	KY678466
*P. subterrestris* A.H. Sm.	HMJAU 37885	Jilin: Changbai Mountain National Nature Reserve	MG734747
*P. subterrestris*	HMJAU 37887	Jilin: Songjiang Town	MG734741

### Data analyses

ITS1+5.8S+ITS2 sequences of 27 species were tested with BLAST in GenBank. Fifty five sequences were downloaded from GenBank, including 21 type species of *Psathyrella*, based on BLAST results and referred to the recent studies (Nagy et al. 2013; von Bonsdorff et al. 2014; Örstadius et al. 2015; Yan and Bau 2017). A total of 103 ITS sequences were aligned using MAFFT 7.205 (Katoh and Standley 2013). The aligned ITS dataset consisted of 643 nucleotide sites (including gaps). The best model (GTR+I+G) was selected by AIC in MRMODELTEST 2.3 (Nylander 2004). Bayesian Inference (BI) was performed with MRBAYES 3.2.6 and four Markov Chains (MCMC) were run for three million generations, sampling every 300th generation. The first 25% trees were discarded (Ronquist and Huelsenbeck 2003). Maximum likelihood analysis was performed with IQTREE 1.5.6 (Nguyen et al. 2014).

## Results

**Figure 1.**
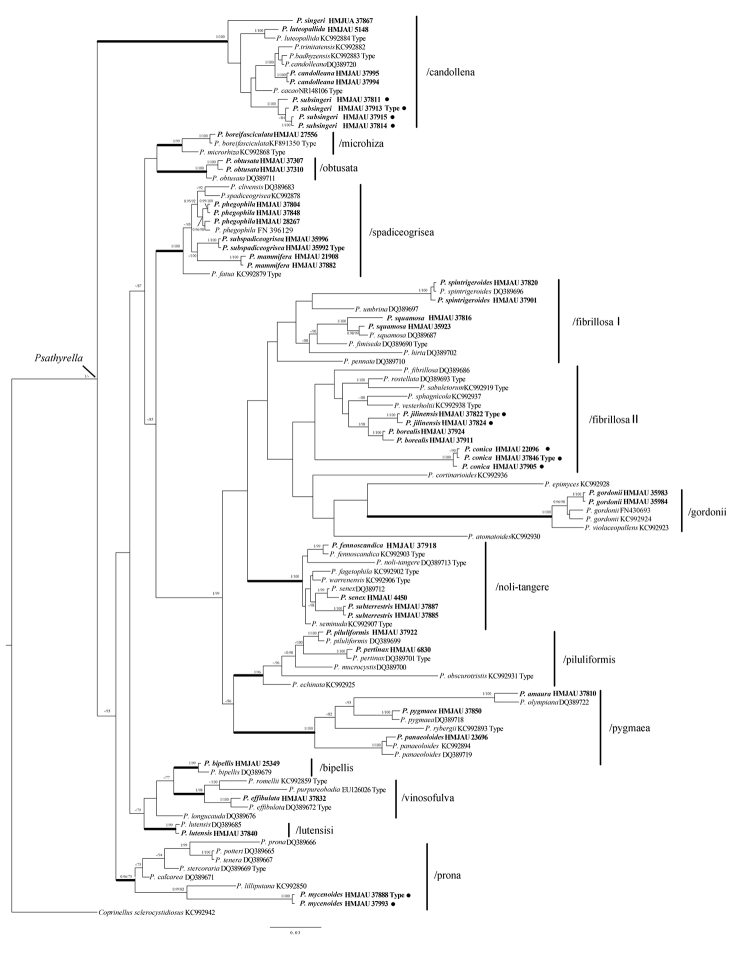
Bayesian and Maximum Likelihood tree inferred from partial ITS sequence data (BPP≥0.95, Bootstrap≥75 are indicated). The tree is rooted with *Coprinellus sclerocystidiosus* (M. Lange & A.H. Sm.) Vilgalys, Hopple & Jacq. Johnson. Newly generated sequences appear in bold. ● indicates newly described species.

The phylogenetic tree (Figure 1) shows that all studied materials fall into *Psathyrella*, with a high statistical support value (BPP=1). It is divided into 14 clades. Most of them have a high statistical support value (BPP≥0.95, Bootstrap≥75), except /fibrillosa I and /fibrillosa II.

Four new species are separated into individual lineages (BPP=1, Bootstrap=100) and are independent from the close taxa. *Psathyrella conica* forms a distinct lineage in /fibrillosa II; *P. jilinensis* belongs to /fibrillosa II and groups together with *P. borealis*; *P. mycenoides* belongs to /prona and is closely related to *P. lilliputana* Örstadius & E. Larss.; and *P. subsingeri* forms a distinct lineage in /candolleana.

The positions of some species are firstly supplemented: *P. amaura* belongs to /pygmaea and is very close to *P. olympiana* A.H. Sm.; *P. borealis* belongs to /fibrillosa II. *P. mammifera* belongs to /spadiceogrisea; *P. singeri* A.H. Sm. belongs to /candolleana; and *P. subterrestris* belongs to /noli-tangere.

### Taxonomy

#### Psathyrella conica

Taxon classificationFungiAgaricalesPsathyrellaceae

T. Bau & J.Q. Yan sp. nov.

823858

Figs 2a–b , 3

##### Diagnosis.

Pileus campanulate to conical, with a subacute to obtuse umbo in early stage. Lamellae 3.0–5.0 mm broad, close. Basidiospores 7.8–8.8 × 4.0–4.5(–5.0) μm, germ pore indistinct or absent. Pleurocystidia numerous, narrowly utriform, with obtuse to broad obtuse or slightly subcapitate at apex. Cheilocystidia scattered.

##### Holotype.

CHINA. Jilin Province, Yanbian Korean Autonomous Prefecture, Antu County, Changbai Mountain, 30 Jun 2017, HMJAU 37846.

##### Etymology.

Name refers to the conical pileus.

**Figure 2.**
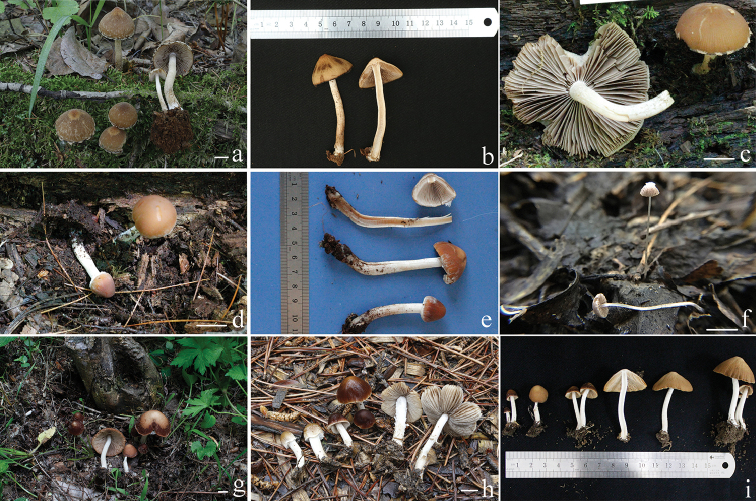
Basidiomata of *Psathyrella* species. **a–b** *Psathyrella conica* **c–e** *Psathyrella jilinensis* **f** *Psathyrella mycenoides* **g–i** *Psathyrella subsingeri*; Bars: 10 mm (**a, c, d, f–h**). Photographs **a–e, g–i** by Jun-Qing Yan; Photograph **f** by Tolgor Bau.

##### Description.

Pileus 12–45 mm, campanulate to conical, with a subacute to obtuse umbo in early stage, hygrophanous, chestnut (7D4–7D6), becoming dirty white with slightly yellowish-brown (6C5–6C6) as drying, striate indistinctly. Veil with a thin coating of white to dirty white (6A1–6B1) fibrils, evanescent. Context dirty white with slightly pink (6B4–6B5), about 3.0 mm thick at stipe centre. Lamellae 3.0–5.0 mm broad, close, adnate to slightly adnexed, coffee-cream (6C4–6C6); edges white (6A1), saw-toothed under 20× magnifier. Stipe 34–85 × 2.0–7.0 mm, cylindrical, slightly expanded or not at base, white, with slightly brown at base, hollow, equal, surface covered with white (6A1) fibrils in early stage, evanescent. Odour and taste indistinctive.

Basidiospores 7.8–8.8 × 4.0–4.5(–5.0) μm, Q=1.8–2.1(–2.3), oblong-ellipsoid to oblong, in profile slightly flattened on one side, pale yellowish-brown in water, yellowish-brown to brown in 5% potassium hydroxide (KOH), inamyloid, smooth, with 1–2 guttulate, germ pore indistinct or absent. Basidia 20–25 × 7.3–9.8 μm, clavate, hyaline, 4- or 2-spored. Pleurocystidia 43–61 × (8.5–)9.8–12 μm, numerous, narrowly utriform, thin-walled, hyaline, with obtuse to broad obtuse or slightly subcapitate, sometimes adhering subhyaline deposits. Cheilocystidia scattered, similar to pleurocystidia, 24–39 × 8.5–12 μm; spheropedunculate or clavate cells abundant, 20–29 × 12–18 μm. Trama of gills irregular, up to 20 μm broad. Pileipellis consisting of 2–3 cells deep layer of subglobose cell, 25–37 μm broad. Clamps present.

##### Habit and habitat.

Solitary to scattered on rotten wood or humus in mixed forests.

##### Other specimens examined.

Jilin Province, Baishan City, Fusong County, Lushuihe town, 7 Jul 2004, HMJAU 4969; 29 Jun 2005, HMJAU 4923; 25 Jun 2009, HMJAU 22096; Yanbian Korean Autonomous Prefecture, Antu County, Changbai Mountain, 23 Jun 2012, HMJAU 25342; 4 Jul 2015, HMJAU 37826; 29 Jun 2017, HMJAU 37847, HMJAU 37904; 6 Aug 2017, HMJAU 37905.

**Figure 3.**
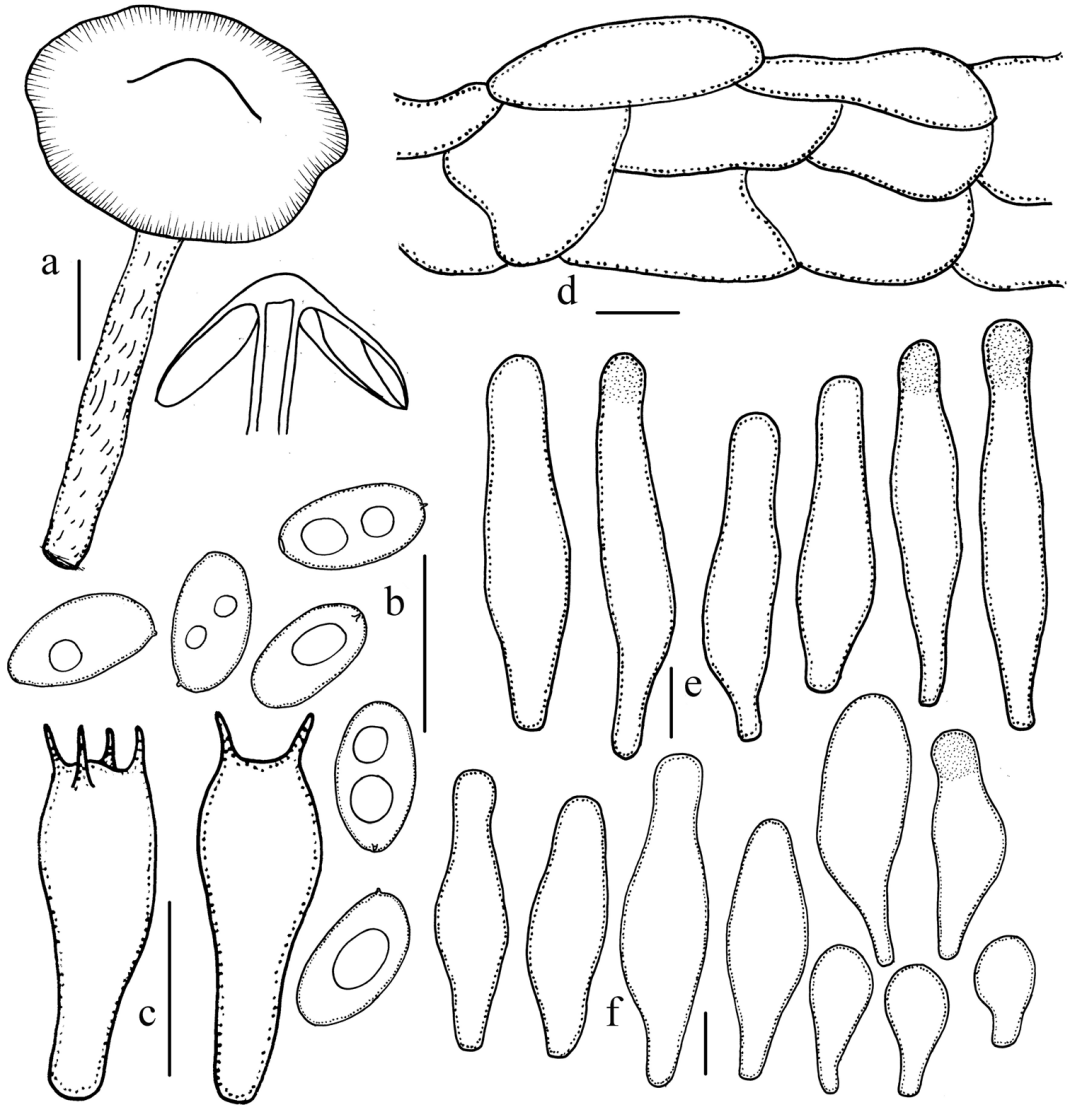
Microscopic features of *Psathyrella conica* (HMJAU 37846). **a** Basidiomata **b** Basidiospores **c** Basidia **d** Pileipellis **e** Pleurocystidia **f** Cheilocystidia. Bars: 10 mm (**a**); 10 μm (**b–f**). Drawing by Jun-Qing Yan.

#### Psathyrella jilinensis

Taxon classificationFungiAgaricalesPsathyrellaceae

T. Bau & J.Q. Yan sp. nov.

823856

Figs 2c–e , 4

##### Diagnosis.

Pileus paraboloid to convex, margin at first appendiculate with adhering patches of white evanescent inner veil. Lamellae 2.0–5.0 mm broad, moderately close. Basidiospores (5.8–)6.3–7.3(–7.8) × (2.9–)3.4–4.4 μm, germ pore absent or indistinct. Pleurocystidia fusiform to narrowly fusiform. Cheilocystidia similar to pleurocystidia. Cheilocystidia and pleurocystidia covered by hyaline, hemispherical amorphous incrustation at apex.

##### Holotype.

CHINA. Jilin Province: Changbai Mountain, Antu County, Yanbian Korean Autonomous Prefecture, 42°23'51"N, 126°05'47"E, 760 m alt., 7 Jul 2015, HMJAU 37822.

##### Etymology.

Name refers to the type locality where the new species was collected.

##### Description.

Pileus 17–45 mm, paraboloid to convex, hygrophanous, reddish-brown (8E5–8E6) at centre, pale yellowish-brown (7C6–7D7) at margin in early stage, yellowish-brown (6B5–6C5), striate up to 1/2 from margin at maturity, becoming slightly brown (7C5–7D6) as pileus dries. Veil white (6A1), thin, fibrillose, at first as appendiculate inner veil or adhering patches at pileus margin, evanescent. Context white (6A1), thin, very fragile, about 2.0 mm thick at centre. Lamellae 2.0–5.0 mm broad, moderately close, adnate, greyish to greyish-brown (7C1–7C3); edges saw-toothed under 20× magnification. Stipe 40–50 × 3.0–7.0 mm, white (6A1), cylindrical, hollow, surface covered with slight white (6A1) evanescent fibrils. Odour and taste indistinctive.

Basidiospores (5.8–)6.3–7.3(–7.8) × (2.9–)3.4–4.4 μm, Q= (1.4–)1.8–2.0(–2.3), oblong-ellipsoid, in profile flattened on one side, pale brown in water, brown in 5% KOH, gradually becoming greyish-brown, inamyloid, smooth, germ pore absent or indistinct, about 0.9 μm wide (if it can be observed). Basidia 15–17 × 6.0–7.0 μm, clavate, hyaline, 4 or 2-spored. Pleurocystidia fusiform, narrowly fusiform, rarely narrowly utriform, thin-walled or slightly thick-walled, apex obtuse to subacute, hyaline, covered by hyaline, hemispherical amorphous incrustation, which can dissolve in 5% KOH. Cheilocystidia 37–51 × 8.5–12 μm, similar to pleurocystidia, hyaline, covered with amorphous incrustation at apex. Trama of gills parallel to hyphae, up to 15 μm broad. Pileipellis consisting of 2–3 cells deep layer of subglobose cell, 20–30 μm broad. Veil composed of cylindrical hyphae, 8.5–10 μm broad. Clamps present.

**Figure 4.**
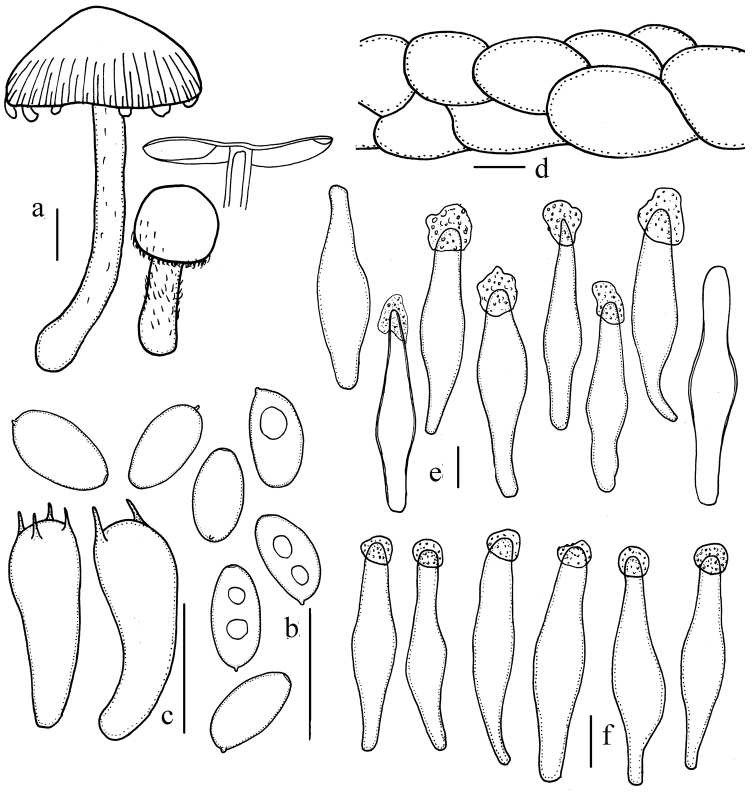
Microscopic features of *Psathyrella jilinensis* (HMJAU 37822). **a** Basidiomata **b** Basidiospores **c** Basidia **d** Pileipellis **e** Pleurocystidia **f** Cheilocystidia. Bars: 10mm (**a**); 10μm (**b–f**). Drawing by Jun-Qing Yan.

##### Habit and habitat.

Solitary to scattered on rotten wood or humus in mixed forests.

##### Other specimens examined.

Jilin Province, Baishan City, Fusong County, Lushuihe town, 27 Jun 2009, HMJAU 22099; 9 Jul 2015, HMJAU 37823; Yanbian Korean Autonomous Prefecture, Antu County, Changbai Mountain, 23 Jun 2012, HMJAU 25351; 31 Aug 2012, HMJAU 25351; Dayangcha, 6 Jul 2015, HMJAU 37824.

#### Psathyrella mycenoides

Taxon classificationFungiAgaricalesPsathyrellaceae

T. Bau sp. nov.

823857

Figs 2f , 5

##### Diagnosis.

Pileus 4.0–5.0 mm, hemispherical to convex. Stipe slender. Basidiospores 8.8–9.2(–9.7) × 4.9–5.4 μm, germ pore distinct, but small. Pleurocystidia scattered, fusiform to lageniform with an obtuse apex. Cheilocystidia lageniform, with an obtuse apex or clavate to spheropedunculate with a long or short mucronate apex.

##### Holotype.

CHINA. Jilin Province, Changchun City, Jilin Agricultural University, 43°48'36"N, 125°24'25"E, 220 m alt., 10 Sep 2016, HMJAU 37888.

##### Etymology.

Name refers to its macroscopic characteristics similar to *Mycena*.

##### Description.

Pileus 4.0–5.0 mm, hemispherical to convex, dirty white with pinkish (7A4–7B5), hygrophanous, striate up to centre from margin. Veil not observed. Context very thin and very fragile, about 0.5 mm thick at stipe centre. Lamellae 1.5–2.0 mm broad, adnate to slightly adnexed, pale brown (7C3–7C4), edges saw-toothed under 20× magnification. Stipe slender, 25–30 × 0.5–1.0 mm, hygrophanous, subhyaline, cylindrical, hollow, equal, fragile, evanescently pruinose at apex.

Basidiospores 8.8–9.2(–9.7) × 4.9–5.4 μm, Q=1.6–2.0, ellipsoid to oblong- ellipsoid, in profile flattened on one side, pale yellowish-brown in water, becoming dark grey to dark brown in 5% KOH, germ pore distinct, but small, about 0.9 μm broad. Basidia 15–17 × 8.8–10 μm, clavate, hyaline, 4- or 2-spored. Pleurocystidia 37–56 × 12–17 μm, scattered, fusiform to lageniform with an obtuse apex, thin-walled and hyaline. Cheilocystidia numerous, 29–44 × 9.8–17 μm, hyaline, lageniform with an obtuse apex or clavate to spheropedunculate, with long or short mucronate apex, rarely spheropedunculate. Trama of gills irregular, hyphae up to 10 μm broad. Pileipellis hymeniderm, cells 20–30 μm broad. Clamps present.

**Figure 5.**
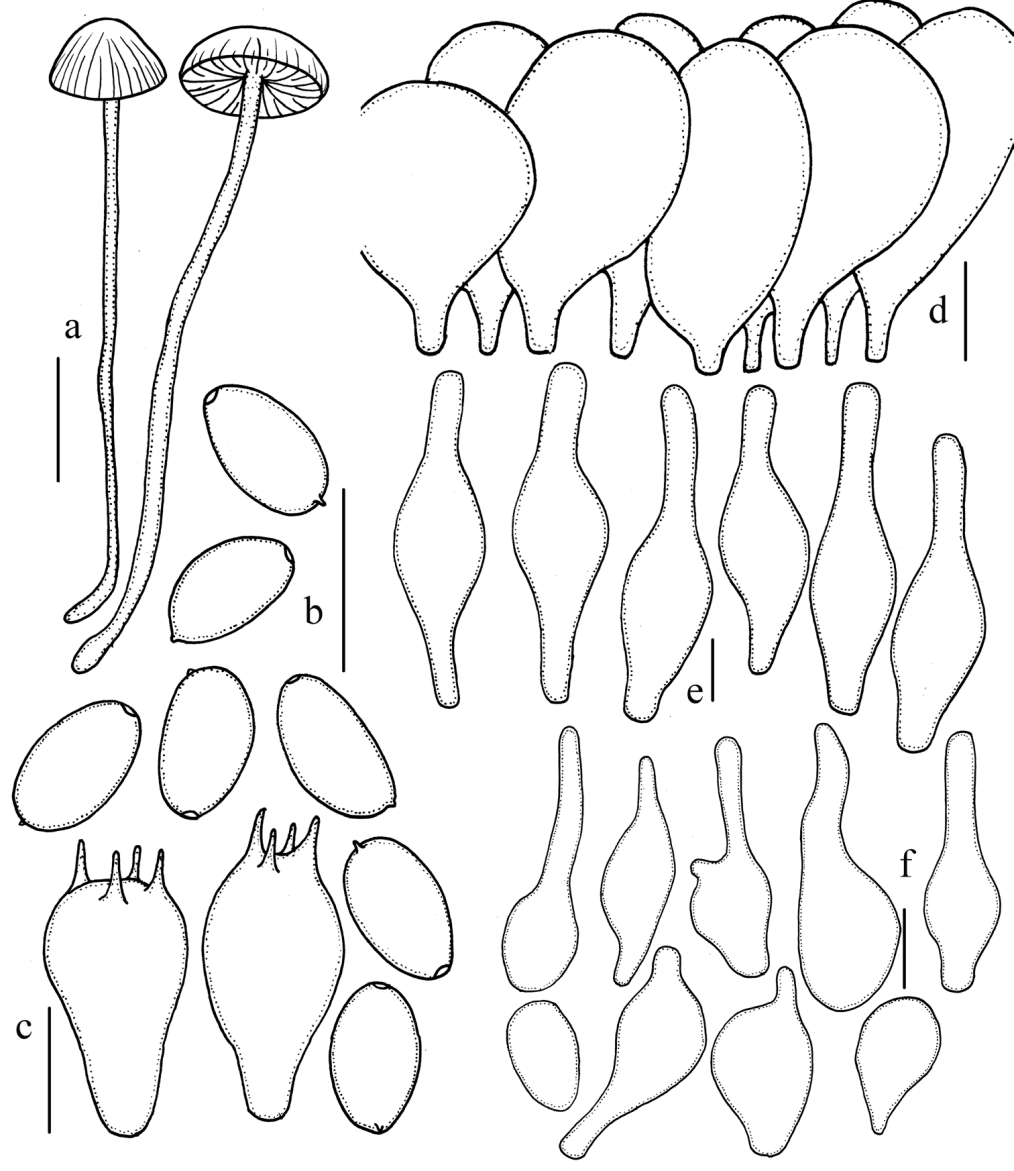
Microscopic features of *Psathyrella mycenoides* (HMJAU 37888). **a** Basidiomata **b** Basidiospores **c** Basidia **d** Pileipellis **e** Pleurocystidia **f** Cheilocystidia. Bars: 10mm (**a**); 10μm (**d–f**). Drawing by Jun-Qing Yan.

##### Habit and habitat.

Solitary to scattered on humus in mixed forests.

##### Other specimens examined.

CHINA. Jilin Province, Changchun City, Jilin Agricultural University, 12 Sep 2016, HMJAU 37993.

#### Psathyrella subsingeri

Taxon classificationFungiAgaricalesPsathyrellaceae

T. Bau & J.Q. Yan sp. nov.

**Figure 6.**
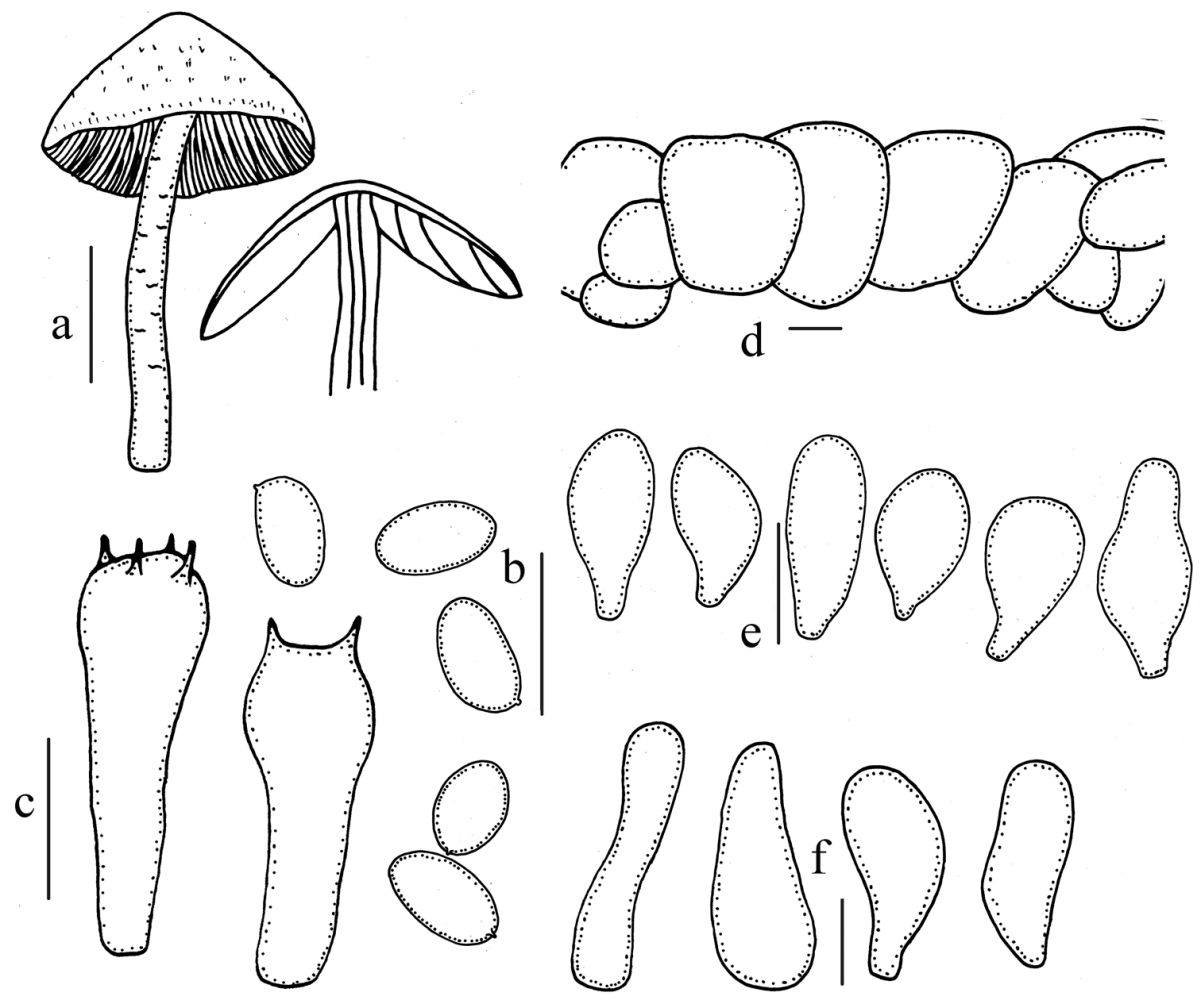
Microscopic features of *Psathyrella subsingeri* (HMJAU 37913). **a** Basidiomata **b** Basidiospores **c** Basidia **d** Pileipellis **e** Cheilocystidia **f** Caulocystidia. Bar: 10 mm (**a**); 10 μm (**b–f**). Drawing by Jun-Qing Yana.

MycoBank: MB823855 Figs 2g–i , 6

##### Diagnosis.

Pileus 15–40 mm, paraboloid to conical. Lamellae 2.0–4.0 mm broad, close. Basidiospores 5.8–7.8(–8.8) × 3.9–4.4(–5.0) μm, very pale, nearly hyaline or slightly yellow in water and 5% KOH. Germ pore absent. Pleurocystidia absent. Cheilocystidia utriform to predominantly spheropedunculate.

##### Holotype.

CHINA. Jilin Province, Changchun City, Jingyuetan National Scenic Area, 43°47'38"N, 125°26'55"E, 200 m alt., 25 Jun 2017, HMJAU 37913.

##### Etymology.

Name refers to its microscopic characteristics similar to *P. singeri*.

##### Description.

Pileus 15–40 mm, paraboloid to conical, obtuse or slightly umbonate at disc, hygrophanous, dark reddish-brown (8E7–8F8) or faint yellowish-brown (5C5–5C4), becoming yellowish-brown (6D5–6D6) as pileus dries, striate indistinct. Veil present in early stage, thin, white (6A1), fibrillose, evanescent. Context white (6A1), thin and very fragile, about 2.5 mm thick at stipe centre. Lamellae 2.0–4.0 mm broad, close, adnate, pale brown (6C4–6C5), edges white (6A1), saw-toothed under 20× magnifier. Stipe 35–50 × 3.0–4.5 mm, cylindrical, hollow, equal, fragile, covered with slight white (6A1) fibrils, which fall off easily. Spore print chocolate (7E7–7E8). Odour and taste indistinctive.

Basidiospores 5.8–7.8(–8.8) × 3.9–4.4(–5.0) μm, Q=1.4–2.0, ellipsoid to oblong-ellipsoid, in profile flattened on one side, very pale, nearly hyaline or slightly yellow in water and 5% KOH, inamyloid, smooth. Germ pore absent. Basidia 15–22 × 7.3–9.8 μm, 4- or 2-spored, clavate, hyaline. Pleurocystidia absent. Cheilocystidia utriform to spheropedunculate, rarely clavate to fusiform with an obtuse to broadly obtuse apex, thin-walled, hyaline. Caulocystidia 26–37 × 9.8–15 μm, rarely, various, clavate, utriform, thin-walled, hyaline. Trama of gills irregular, up to 15 μm broad. Pileipellis consisting of 1–2 cells, deep layer of subglobose cell, 20–32 μm broad. Clamps present.

##### Habit and habitat.

Solitary to scattered on terrestrial or humus in mixed forests.

##### Other specimens examined.

Henan Province, Xinyang City, Boerdeng National Forest Park, 16 Jul 2017, HMJAU 37915; Xian Mountain, 15 Jul 2017, HMJAU 37931; Jilin Province, Changchun City, Jilin Agricultural University, 21 Jun 2016. HMJAU 37811; Jingyuetan National Scenic Area, 25 Jun 2017, HMJAU 37914; 7 Jul 2017, HMJAU 37849; Tonghua City, Qiupi Village, 6 Aug 2015, HMJAU 37812, HMJAU 37813; Yunnan Province, Yeya Lake, 7 Aug 2016, HMJAU 37814; 6 Aug 2017, HMJAU 37852; 23 Aug 2017, HMJAU 37962.

## Discussion

These phylogenetic results are very much in congruence with the study of Larsson and Örstadius (2008) and Örstadius et al. (2015), except /fibrillosa, which separates to two lineages (/fibrillosa I and /fibrillosa II). As only ITS sequences were analysed in this study, this accounts for the difference and the very low support value (BPP<0.3). Four new species are separated into individual lineages (BPP=1) and distinct from other closely related taxa.

*Psathyrella conica* is a distinct lineage in fibrillosa II, which is independent from any other related taxa. Morphologically, it can be classified in subsection Spadiceogriseae (Kits van Waveren 1985). Only *P. clivensis* (Berk. & Broome) P. D. Orton does not have a germ pore in this subsection, but basidiospores of *P. clivensis* are obviously broader, 8–10 × 5.5–6.5 μm and ellipsoid to ovoid (Kits van Waveren 1985). It can also be classified in section Fatuae (Smith 1972), some species having sturdy stipe and utriform cystidia, but they can be clearly distinguished from *P. conica* by other micromorphology. *Psathyrella acadiensis* A.H. Sm. has smaller basidiospores, which are only up to 6.0 μm long; *P. albocinerascens* A.H. Sm. has an obvious germ pore and white pileus in the early stage; *P. amarella* A.H. Sm. and *P. spadiceogrisea* (Schaeff.) Maire have an obvious germ pore; *P. vesiculocystis* A.H. Sm. has pedicellate-pleurocystidia (Smith 1972). Furthermore, *P. terrestris* Natarajan has aspects of *P. conica*, whose pileus is umbonate, but it has broadly utriform pleurocystidia and its basidiospores are dark brown, subglobose and up to 8.5 μm broad (Natarajan 1978).

*Psathyrella jilinensis* grouped together with *P. borealis* in /fibrillosa II. However, *P. borealis* has an obvious germ pore. Morphologically, it can be classified in section Hydrophilae by basidiospores rarely exceeding 7.5 μm and the presence of pleurocystidia. There are hardly any other species in the section that match the characteristics of *P. jilinensis*. The pleurocystidia of *P. atomatoides* (Peck) A.H. Sm. do not have amorphous incrustation. Basidiospores of *P. cortinarioides* P.D. Orton and *P. pertinax* have a clearly truncated base. Cystidia of *P. umbrina* Kits van Wav. have subacute apex and their basidiospores are broader, up to 4.5–5.5 μm (Kits van Waveren 1985; Örstadius and Kundsen 2012). Furthermore, *P. cokeri* (Murrill) A.H. Sm., *P. pennata* and *P. subsimilissima* A.H. Sm. have some similar aspects of *P. jilinensis*, but *P. cokeri* (Murrill) A.H. Sm. and *P. subsimilissima* A.H. Sm. do not have amorphous incrustation (Smith 1972) and *P. pennata* grows on burnt soil, its basidiospores being larger and narrowly amygdaloid (Örstadius and Kundsen 2012).

*Psathyrella mycenoides* belongs to /prona and is placed close to *P. lilliputana*. However *P. lilliputana* has larger (9.5–11 × 5.0–6.0 μm) and snout-like basidiospores (Örstadius et al. 2015). Morphologically, more than 10 species of *Psathyrella* have very small basidiomata, whose pileus rarely exceeds 10 mm, but they can be separated by obvious characteristics as follows: *P. byssina* (Murrill) A.H. Sm. and *P. scheppingensis* Arnolds have smaller basidiospores, which rarely exceed 7.5 μm (Smith 1972; Arnolds 2003); *P. coprinoides* A. Delannoy, Chiaffi, Courtec. & Eyssart. and *P. tenuicula* (P. Karst.) Örstadius & Huhtinen have pileocystidia and slender basidiospores (Örstadius and Huhtinen 1996; Delannoy et al. 2002); the coprophilous fungi of *P. granulose* Arnolds have utriform cystidia (Arnolds 2003); basidiospores of *P. liciosae* Contu & Pacioni are partly phaseoliform in side view and ochraceous-brown in 5% KOH (Contu and Pacioni 1998); *P. minima* Peck has very distant lamellae (Peck 1878); and basidiospores of *P. psilocyboides* A.H. Sm. are truncated at the base (Smith 1972).

*Psathyrella subsingeri* belongs to /candollena. Only *P. luteopallida* and *P. singeri* have nearly hyaline basidiospores pores in this clade. However, the basidiospores of *P. luteopallida* are longer than 8.0 μm. The basidiospores of *P. singeri* are broader, up to 5.5 μm (Smith 1972). Morphologically, *P. subsingeri* belongs to section Spintrigerae with basidiospores less than 9.0 μm and absent pleurocystidia (Kits van Waveren 1985). Its cheilocystidium is similar to *P. submicrospora* Heykoop & G. Moreno [= *Coprinopsis submicrospora* (Heykoop & G. Moreno) Örstadius & E. Larss.], but basidiospores of *P. submicrospora* are predominantly amygdaliform (Heykoop and Moreno 2002). It also can be classified in series *Atricastaneae* (Smith 1972). There are only three species in the series that match the characteristic of subhyaline to hyaline basidiospores in water or 5% KOH. However, they can be separated as follows: the basidiospores of *P. atricastanea* (Murrill) A.H. Sm are truncate; *P. albipes* A.H. Sm. and *P. subhyalinispora* (Murrill) A.H. Sm. differ in having an obvious germ pore (Smith 1972). Furthermore, *P. aequatoriae* Singer has subhyaline to hyaline basidiospores, but differs by smaller and sometimes papillate pileus. *Psathyrella olympiana* and *P. bipellis* [= *P. odorata* (Peck) Sacc.] have aspects of *P. subsingeri* in macroscopic characteristics, whose pileus are reddish-brown, but have pleurocystidia (Örstadius and Kundsen 2012).

### Key to species of *Psathyrella* in Northeast China

**Table:** 

1	Pleurocystidia absent	**2**
–	Pleurocystidia present	**5**
2	Basidiospores brown in 5% KOH	***P. candolleana***
–	Basidiospores very pale, subhyaline in 5% KOH	**3**
3	Basidiospores predominantly longer than 8.0 μm	***P. luteopallida***
–	Basidiospores shorter	**4**
4	Basidiospores up to 5.5 μm broad	***P. singeri***
–	Basidiospores up to 4.5 μm broad	***P. subsingeri***
5	Basidiospores longer than 10 μm, pleurocystidia utriform to clavate, sometimes with yellowish-brown inclusions	***P. bipellis***
–	Not as above	**6**
6	Germ pore always or predominantly distinctly visible	**7**
–	Germ pore absent or predominantly indistinctly visible	**19**
7	Pleurocystidia rarely, lageniform, shorter than 40 μm, clamps absent	***P. effibulata***
–	Not as above	**8**
8	Basidiospores up to 7.0 μm long	**9**
–	Basidiospores longer	**10**
9	Pleurocystidia up to 35 μm long, mostly with distinct crystals	***P. pygmaea***
–	Pleurocystidia up to 65 μm long, without crystals	***P. piluliformis***
10	Basidiomata densely caespitose, cheilocystidia fusiform or mucronat, basidiospores 8.5–9.8 × 4.6–5.1 μm	***P. boreifasciculata***
–	Not as above	**11**
11	Pleurocystidia mostly fusiform or lageniform	**12**
–	Pleurocystidia mostly utriform, narrowly utriform or clavate	**14**
12	Basidiomata vary small, pileus up to 5.0 mm, cheilocystidia with long or short mucronate	***P. mycenoides***
–	Not as above	**13**
13	Grown on sphagnum, basidiospores 8.8–9.2 × 4.4–5.0 μm	***P. borealis***
–	Terrestrial or basidiomata attached to bits of woody debris, basidiospores 7.3–8.8 × 4.1–4.4 μm	***P. subterrestris***
14	Basidiospores distinctly triangular	***P. panaeoloides***
–	Not as above	**15**
15	Pleurocystidia often with in ammonia greenish deposits, basidiospores 8.8–9.7 × 4.4–4.9 μm	***P. lutensis***
–	Not as above	**16**
16	Cheilocystidia clavate to spheropedunculate, pleurocystidioid cheilocystidia scattered	***P. phegophi la***
–	Pleurocystidioid cheilocystidia numerous	**17**
17	Basidiospores up to 6.0 μm broad	***P. fennoscandica***
–	Basidiospores up to 5.0 μm broad	**18**
18	Veil strongly developed and flocculose, pleurocystidia utriform or clavate	***P. gordonii***
–	eil with a thin coating of fibrils, pleurocystidia narrowly utriform	***P. senex***
19	Pleurocystidia distinctly thick-walled or slightly thick-walled, covered with distinct crystals, basidiospores up to 9 μm long	**20**
–	Not as above	**21**
20	Pleurocystidia utriform, distinctly thick-walled	***P. amaura***
–	Pleurocystidia fusiform, slightly thick-walled or thin-walled	***P. jilinensis***
21	Pleurocystidia fusiform, lageniform, with obtuse or subacute apex	**22**
–	Pleurocystidia utriform, narrowly utriform, with obtuse to broad obtuse apex	**25**
22	Cheilocystidia mucronate, basidiospores ellipsoid, pale brown in 5% KOH	***P. obtusata***
–	Not as above	**23**
23	Basidiospores (6.8–)7.3–7.8(–8.8) × 3.4–4.9 μm, base often broadly truncate, in profile often phaseoliform	***P. pertinax***
–	Not as above	**24**
24	Basidiospores oblong to oblong-ellipsoid, pleurocystidia thin-walled	***P. squamosa***
–	Basidiospores ellipsoid, pleurocystidia slightly thick-walled	***P. spintrigeroides***
25	Basidiospores reddish-brown in water	***P. mammifera***
–	Basidiospores yellowish-brown or pale yellowish-brown in water	**26**
26	Pileus often with subacute or obtuse umbo, basidiospores 7.8–8.8 × 4.0–4.5(–5.0) μm, oblong to oblong-ellipsoid	***P. conica***
–	Pileus without umbo, basidiospores 6.8–7.8 × 3.9–4.9 μm, ellipsoid, rarely oval	***P. subspadiceogrisea***

## Supplementary Material

XML Treatment for Psathyrella conica

XML Treatment for Psathyrella jilinensis

XML Treatment for Psathyrella mycenoides

XML Treatment for Psathyrella subsingeri
